# Impact of interprofessional education about psychological and medical comorbidities on practitioners’ knowledge and collaborative practice: mixed method evaluation of a national program

**DOI:** 10.1186/s12913-016-1720-z

**Published:** 2016-09-02

**Authors:** Christine B. Phillips, Sally Hall, Michelle Irving

**Affiliations:** 1 Social Foundations of Medicine, Medical School, Australian National University, 54 Mills St, Canberra, 0200 Australia; 2 Rural Clinical School, Medical School, Australian National University, Canberra, Australia

**Keywords:** Continuing education, Chronic disease, Team, Mental health, Interprofessional education

## Abstract

**Background:**

Many patients with chronic physical illnesses have co-morbid psychological illnesses, which may respond to interprofessional collaborative care. Continuing education programs frequently focus on skills and knowledge relevant for individual illnesses, and unidisciplinary care. This study evaluates the impact of “Mind the Gap”, an Australian interprofessional continuing education program about management of dual illnesses, on practitioners’ knowledge, use of psychological strategies and collaborative practice.

**Methods:**

A 6-h module addressing knowledge and skills needed for patients with physical and psychological co-morbid illnesses was delivered to 837 practitioners from mixed health professional backgrounds, through locally-facilitated workshops at 45 Australian sites. We conducted a mixed-methods evaluation, incorporating observation, surveys and network analysis using data collected, before, immediately after, and three months after training.

**Results:**

Six hundred forty-five participants enrolled in the evaluation (58 % GPs, 17 % nurses, 15 % mental health professionals, response rate 76 %). Participants’ knowledge and confidence to manage patients with psychological and physical illnesses improved immediately. Among the subset surveyed at three months (response rate 24 %), referral networks had increased across seven disciplines, improvements in confidence and knowledge were sustained, and doctors, but no other disciplines, reported an increase in use of motivational interviewing (85.9 % to 96.8 %) and mindfulness (58.6 % to 74 %).

**Conclusions:**

Interprofessional workshops had an immediate impact on the stated knowledge and confidence of participants to manage patients with physical and psychological comorbidities, which appears to have been sustained. For some attendees, there was a sustained improvement in the size of their referral networks and their use of some psychological strategies.

**Electronic supplementary material:**

The online version of this article (doi:10.1186/s12913-016-1720-z) contains supplementary material, which is available to authorized users.

## Background

Chronic physical illnesses frequently co-exist with psychological illnesses [1]. Chronic illnesses and psychological illnesses can each escalate progression of the other, and each impacts on patients’ self-management capacities. The standard medications for many psychiatric illnesses have metabolic side-effects, just as some medications for chronic illnesses can have psychological side-effects. The general practice consultation may be the best opportunity for many patients with co-morbid psychological and physical illness to be identified and commence treatment [2]. However, incident depression in patients with chronic disease [3, 4], and chronic disease in those with psychological illness are often under-detected in primary care [5, 6].

One of the challenges for clinicians working with co-morbid psychological and physical illnesses is that the evidence base for best practice [7, 8], and continuing education programs, tend to focus on individual illnesses in isolation. In practice, clinicians cannot abstract decisions about treatment of one illness from consideration of the patient’s other illnesses.

Patients with co-morbidities often experience uncoordinated care from a porous network of clinicians and services [9, 10]. Collaborative care models can improve clinical outcomes for patients with a range of chronic diseases and depression [11–13]. A number of Medicare-subsidised items exist in Australia that could support more interdisciplinary care for patients with co-morbid psychological and physical illnesses. Patients with chronic disease can access chronic disease management and team care items [14] under Medicare, as well as subsidised referrals to some allied health practitioners. Patients with psychological illnesses can be referred for a set number of Medicare-subsidised consultations from mental health professionals in the private sector, under the Better Access initiative [15]. All these policy initiatives rely on health professionals having a suite of management approaches for both physical and psychological illnesses, and the ability to collaborate with one another on a joint plan.

More than two-thirds of treating psychologists in Australia work in private practice [16]. Their connections with GPs have historically been patchy, since patients can self-refer to both disciplines, and there has been no obligation for the professionals to communicate with each other. Even though there has been good take-up of the Better Access Medicare items, these appear to be mainly accessed by people under the age of 45 years, rather than the older age groups more likely to suffer from co-morbid physical illnesses [17]. The most recent review of the Better Access initiative called for more pathways between GPs and psychologists [18]. Interprofessional education (IPE) offers the potential for disciples to learn perspectives shared by one another, and to become better collaborators. Although IPE has proven benefits in developing knowledge and skills in individual practitioners, there are few studies exploring organisational changes or changes in collaborative practice with other health professionals [19]. How to effectively develop and deliver educational programs addressing co-morbidity also receives little attention in the evaluation literature [20].

This paper aims to contribute to knowledge in both of these areas. We report immediate knowledge, and longer term skills and collaborative practice outcomes of “Mind the Gap”, a national interprofessional educational program for allied health professionals, GPs and mental health professionals, addressing the care of patients with co-morbid psychological and chronic physical illnesses.

## Methods

### Intervention

Mind the Gap was an advanced learning module which aimed to develop participants’ skills and knowledge to work singly or interprofessionally with patients with co-morbid psychological and physical illnesses. Aims, content and educational strategies are summarised in Table 1. The module was delivered in one six-hour workshop, or two three-hour workshops, facilitated by a local clinician with expertise in psychological care. All facilitators received a facilitator’s guide and a presentation, with speaking notes. The program was delivered through the Medicare Locals, primary care support organisations, which at the time of the study had 61 regional offices across Australia. As this program was funded by the Department of Veterans’ Affairs, one of the referral pathways covered was to the Veterans and Veterans Families Counselling Service (VVCS), an Australian Government funded service providing counselling and support for war and defence service-related mental health conditions [21].

**Table 1:** Learning objectives, domain of learning and educational strategies used in the Mind the Gap advanced learning module

Learning objective	Domain of learning	Educational strategy
Increase understanding of the aetiology, epidemiology, and interrelationships of psychological and chronic medical illnesses	Knowledge	Summary of existing research presented in accessible format by facilitator with back-up reading material
Increase understanding of patient experiences of co-morbid psychological and chronic medical illnesses	Knowledge Sensitivity	Personal presentation by carer or consumer from the local region about their experiences with the health sector, and managing co-morbid illnesses. Reflective exercise between participants.
Develop knowledge and skills in assessment of co-morbid psychological and chronic medical illnesses	Clinical skills, organisational changes	Practise using assessment tools, management and relapse prevention strategies. Case study exploring case management. Distribution of materials on use of practice systems to enhance continuity of care
Recognise the roles and skills of mental health and allied health practitioners in treatment of co-morbid psychological and chronic medical illnesses	Attitudes Collaborative practice	Inter-professional case study discussion. Exercise identifying local inter-professional referral pathways
Become conversant with strategies and treatments suitable for co-morbid psychological and chronic medical illnesses in general practice populations	Clinical skills Confidence	Introduce basic principles of mindfulness, behavioural activation, motivational interviewing, solution-focused therapy, relaxation strategies, and dealing with grief. Group exercise in mindfulness; facilitated reflection on video example of motivational interviewing

### Program design

The theory of instructional design underpinning the Mind the Gap learning module reflects Reigeluth’s Elaboration Theory [22]: that is, that effective teaching about complex topics requires the information to be reconfigured into smaller units of information which are scaffolded in order to accommodate learning and memory limitations. Recognition and management of co-morbid psychological and medical conditions is a complex topic. Most clinicians are likely to have some prior knowledge or pre-conceptions about this topic, which may be called upon to act as scaffolding for deeper learning. The instructional design therefore began with familiar information, drawing on the statistics of co-morbidity, encouraging clinicians to contextualize this in their own clinical experiences. The less familiar material – counselling techniques, referral processes – were then sequenced and arranged around more familiar concepts, such as one’s own referral networks.

### Evaluation design

**Fig. 1:**
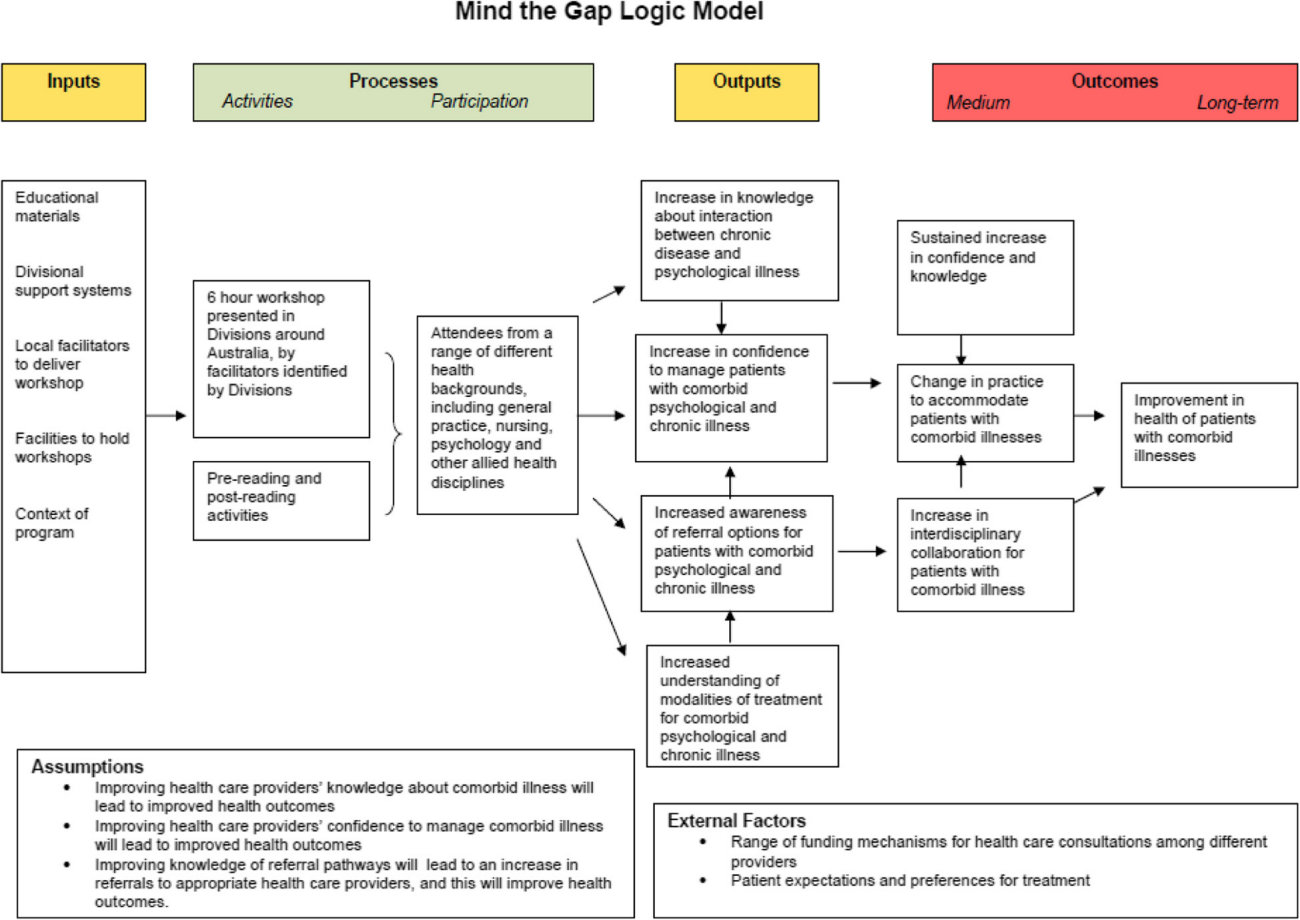
Mind the Gap Educational Program Logic Model

The evaluation aimed to (1) assess the effectiveness of information delivery and workshop facilitation; (2) measure change in knowledge and attitudes among participants; and (3) assess the impact of the learning module on provider behaviour, including application of newly acquired skills in clinical practice and on interprofessional collaboration. This was a systems based evaluation examining inputs, processes, outputs and outcomes [23]. *Process indicators* collected were: satisfaction with the delivery and design of the program. *Output indicators* were: immediate changes in knowledge, attitudes to interprofessional practice, and confidence to undertake psychological strategies with patients with physical and psychological comorbidities. *Outcome indicators* (sought at three months after the intervention) prioritised in this evaluation were: sustained application of skill-sets for patients with co-morbid psychological and chronic physical illnesses; changes in organisational practices; and increase in interprofessional collaboration (assessed through social network analysis). Figure 1 presents the logic model of the educational program, used to frame the evaluation.

### Data collection

Participants completed two questionnaires at baseline and three months after the workshop: a 27 item questionnaire addressing confidence, knowledge and skills used in managing patients with co-morbid illness, and attitudes to other professionals (Additional file 1: Figure S1); and a 15-item social network questionnaire exploring the size and structure of the individual’s networks with other health professionals (Additional file 2: Figure S2).

The 27-item questionnaire comprised the Interprofessional Socializing and Valuing Scale (ISVS) [24], measured on a seven-point Likert scale, and a number of items about participants’ knowledge, experience and confidence managing patients with comorbid illnesses, measured on a five-point Likert scale.

The 15-item social network questionnaire explored ego (personal) networks, the networks centred on individual practitioners [25]. Participants were asked to rate relationships with a total of 15 health professionals identified as being of particular relevance to management of co-morbid chronic disease and psychological illness; they could also nominate up to 6 additional professionals. Additional file 3: Table S1 shows the range of disciplines identified by participants in this study. The survey collected data on three different professional relationships within each personal network: the sharing of information, referral patterns, and working together in other ways. Data were paired using an anonymised unique identifier.

Participants were also asked to complete a 10-item questionnaire immediately after the workshop, addressing confidence or knowledge, and their plans (if any) to change using a commitment to change (CTC) [26] approach (Additional file 4: Figure S3). CTC statements from participants focused on intended changes as a result of training (maximum of three). The 3 month follow-up survey (Additional file 5: Figure S4) also included some open questions on whether the changes had been achieved and were likely to be continued, and challenges in instituting the change. The degree of difficulty in instituting changes was rated on a five-point Likert Scale.

Four workshops were observed by three evaluators, using a structured observation sheet (total observation, 23 h).

### Distribution of surveys

**Fig. 2:**
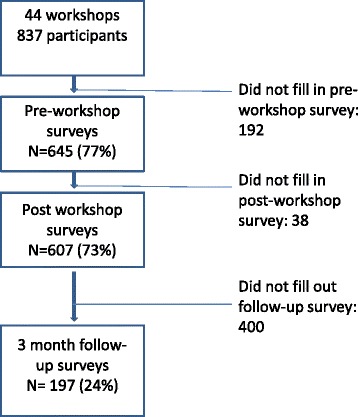
Response rates at baseline, immediately after, and three months after the workshop

Pre and post questionnaires were distributed to participants at workshops by facilitators. Participating Medicare Locals facilitated distribution and follow-up of questionnaires three months after each workshop. Pre and post data were provided for 41 workshops, and follow up data were provided by the Medicare Locals for participants from 30 workshops. Figure 2 presents the response rates at each sampling interval.

### Analysis

Pre and post workshop data were analysed using SPSS (v20.0, IBM). As a quality assurance measure, data entries from surveys collected from 9 % of the workshops were double-checked by a second evaluator (concordance 96 %). Changes in knowledge, awareness, confidence and attitudes were assessed by treating Likert scales as continuous data, once it had been confirmed that the means were normally distributed. Matched pairs analysis using t-tests was conducted for before-after, and before-follow up groups. Participants did not all use their unique identifying codes on all three surveys, so comparisons were not made across all three groups. Use of different psychological strategies at follow-up was stratified by discipline group, to accommodate a slight over-representation of doctors in the three-month follow-up data. Commitment to change data were compared to pre-workshop and 3 month follow up data using matched samples. Network analysis was performed using UCINET [27] software, comparing network characteristics before and after the intervention. Social network derived data were also entered into an SPSS-X compatible dataset and analysed using a paired samples analysis where indicated.

## Results

Forty-five workshops were held across seven states and territories, with between 10 and 54 attendees (mean, 19) per workshop. Of the 837 workshop participants, 645 enrolled in the evaluation (54 % GPs, 16 % nurses, 14 % mental health professionals, response rate 77 %). Other allied health practitioners who attended were occupational therapists, pharmacists, physiotherapists, podiatrists and social workers. At three-month follow-up, doctors were over-represented (64 %), and mental health workers and nurses relatively under-represented (11 % and 6 % respectively).

Workshops observed in several states were consistent in their delivery of the program as designed, with the same materials being used in all of them. The workshops were generally well received. Of 590 who provided feedback, 75 % felt that the five learning objectives had been fully met.

### Improvement in knowledge and confidence

Immediately after the workshop, improvements were reported across all six knowledge and confidence items. The subsample who completed the three month evaluation reported higher mean scores for each item than the overall mean at baseline (Table 2). All these differences are statistically significant, reflecting the large sample size, with changes in knowledge being greater than changes in confidence.

**Table 2:** Matched-pairs analysis of practitioners’ knowledge and confidence to treat patients with physical and psychological illnesses

	Before Mean (sd) (*n* = 522)	After^a^ Mean (sd) (*n* = 522)	At three months^b^ Mean (sd) (*n* = 160)
Knowledge			
Knowledge of patient self-management approaches and strategies	3.18 (0.72)	4.02 (0.63)	3.96 (0.76)
Knowledge of how to assess co-morbid chronic illness and psychological illness	3.12 (0.82)	4.06 (0.69)	4.06 (0.68)
Knowledge of management planning for co-morbid chronic illness and psychological illness	2.85 (0.97)	3.95 (0.84)	3.96 (0.84)
Knowledge of relapse prevention strategies planning	2.90 (0.83)	3.84 (0.73)	3.89 (0.64
Knowledge of consumer perspectives and carer experiences	2.98 (0.77)	4.05 (0.68)	3.92 (0.63)
Confidence			
Confidence to recognise patients with comorbid physical and psychological illnesses	3.39 (0.70)	4.08 (0.75)	4.06 (0.51)
Confidence in using psycho-educational strategies	2.88 (0.92)	3.81 (0.84)	3.77 (0.79)
Confidence in integrating therapies	2.96 (0.96)	3.74 (0.93)	3.79 (0.80)
Confidence about meeting the needs of carers	3.01 (0.78)	3.85 (0.75)	3.77 (0.66)

^a^P value for all categories, comparison of baseline and post-workshop : *p* < 0.0001

^b^P value for all categories, comparison of baseline and 3 month follow-up: *p* < 0.0001

### Increase in use of psychological strategies

Mental health professionals and social workers reported nearly 100 % use of the strategies taught at this workshop at baseline, and at three-month follow-up. When analysed by professional group, the group that reported the most significant change in uptake of strategies was GPs, who had significant increases in reported use of mindfulness and motivational interviewing (Table 3).

**Table 3:** Psycho-educational techniques used by doctors, before and three months after Mind the Gap training workshop

Techniques used at least once in the preceding three months	Baseline *n* = 348	Three months *n* = 127
Motivational Interviewing	85.9 %	96.8 %^a^
Behavioural activation	73.0 %	79.5 %
Solution focused therapy	76.7 %	82.7 %
Mindfulness	58.6 %	74.0 %^b^
Relaxation	81.6 %	89.0 %
Grief and loss counselling	80.4 %	85.1 %

^a^ *X* ^2^ = 10.14; *p* = 0.001

^b^ *X* ^2^ = 9.403; *p* = 0.002

### Changes in clinical practice

Five hundred ten participants provided responses to this question at baseline. Narrative analysis indicated that the following intentions to change were described:

- Actively seeking and assessing patients for physical and psychological co-morbidities.
- Incorporating specific scales and tools into routine practice (eg DASS-21 [28], or the Geriatric Depression Scale [29])
- Improved care planning and shared management, including better collaboration with local services
- Involving patients and carers more in disease management planning

One hundred fifty-three participants provided usable responses to the follow-up questionnaire on changes to practice. Respondents indicated that changes instituted were the same as planned changes in 76 % (322) of 421 changes. Matched data between post and follow-up workshop questionnaires were available for 130 respondents from 30 workshop sites, reflecting on 369 changes, or which 77 % were achieved. For this group, changes proposed post-workshop and those instituted and reported at follow up were broadly consistent although often varied in detail. Actual changes in practice tended to be more specific and incremental, for example “try to undertake more chronic disease management” became “I now schedule more time with complex patients” or “collaborating better with local GPs” became “including more detail in letters to doctors”. Respondents who had instituted changes suggested that practice changes had been easy or very easy to make in 61 % of cases. In 22 % (62 changes) they were considered difficult to implement. There were no significant differences between participant discipline or type of change across those changes rated as hard and those rated as easy, suggesting that local or individual factors determined the ease of instituting change.

### Change in attitude to interprofessional working

At baseline, respondents’ scores indicated they had been in agreement with all statements. In the matched pairs analysis, there was improvement in the three month follow-up subsample (Table 4).

**Table 4:** Matched-pairs analysis of interprofessional socialization and valuing scale (ISVS), before and three months after workshops

	Before Mean (sd) (*n =* 154)	At three months Mean (sd) (*n* = 154)	p
I feel comfortable initiating discussions about sharing responsibility for client care	4.48 (1.38)	5.34 (1.20)	<0.00001
I am comfortable engaging in shared decision making with clients	4.63 (1.50)	5.40 (1.38)	<0.00001
I feel comfortable clarifying misconceptions about the role of someone in my profession	4.57 (1.60)	5.43 (1.17)	<0.00001
I see myself as preferring to work on an inter-professional team	4.82 (1.53)	5.41 (1.42)	<0.00001
I am comfortable being the leader in a team situation	4.27 (1.66)	4.87 (1.46)	<0.00001
I feel confident taking on different roles in a team	4.45 (1.60)	5.19 (1.48)	<0.00001
I feel comfortable speaking out within the team when others are not keeping the client’s best interests in mind	4.75 (1.57)	5.44 (1.34)	<0.00001
I believe that inter-professional practice is difficult to implement	3.58 (1.58)	3.59 (1.65)	0.959

### Change in collaborative practice

Five hundred and forty-one participants (65 %) responded to the pre‐workshop social network questionnaire and follow up data were available from 166 respondents from 27 (60 %) workshop locations. Matched data were available for 116 respondents. There were no significant changes in the size of respondents’ professional networks either overall or for each type of relation (information exchange, referral or collaboration) (Table 5). There were also no significant differences in total number of network ties, directionality of information and referral flow, or frequency of interaction within networks, between baseline and follow up.

**Table 5:** Size of different network types, at baseline and three months, matched pairs analysis (*n* = 116)

Network type	Mean size at baseline	Mean size at 3 months	*P* value
Professional network	10.66	10.48	.589
Information exchange	9.92	9.78	.706
Referral	9.76	9.62	.685
Collaboration	8.03	7.79	.559

There was an increase in total number of network connections between respondents and four specific professional disciplines. Significant increases occurred in network ties to exercise physiologists, psychologists, psychiatrists and the Veterans and Veterans Families Counselling Service (VVCS) [21] (Table 6). Network ties to other counsellors and cardiologists significantly decreased over the same period. Respondents also described a significant increase in mean frequency of interactions with psychologists (+0.074, *p* = 0.016), the only group for which this was the case.

**Table 6:** Statistically significant changes in respondents network ties by discipline, matched pairs analysis (*n* = 116)

Professional Discipline	Mean number of ties per network type			Proportion of respondents with any relationship (%)			*p value*
	Pre	3 months		Pre	3 months		
Exercise Physiologist	48	53	+5	51.7	56.0	+4.3	0.015
Psychologist	90	98	+8	83.6	92.2	+8.6	0.006
VVCS	19	23	+4	19.0	25	+6.0	0.012
Psychiatrist	79	85	+6	81.0	85.3	+4.3	0.0002
Other counsellors	62	57	−5	58.6	56.9	−1.7	0.044
Cardiologist	63	56	−7	65.5	57.8	−7.7	0.008

Outgoing referrals were the most common type of network tie overall, with incoming referrals the least common. This remained constant at baseline and at follow-up (Additional file 6: Table S2). By individual discipline, respondents consistently indicated the strongest type of tie with practice nurses was collaboration, and with pharmacists, seeking information.

## Discussion

The interprofessional workshops had an immediate impact on the stated knowledge and confidence of participants to manage patients with physical and psychological comorbidities. For participants for whom follow-up data was available, there was a sustained improvement in stated knowledge and confidence and in their use of some psychological interventions.

The collaborative practice outcomes were less marked. There was a sustained improvement in attitudes to inter-professional recorded at three months. The increase in actual networking was most marked between doctors and psychologists, perhaps reflecting the fact that these were the two largest participant groups. In this regard, “Mind the Gap” met its objectives of improving attitudes and pathways between some primary care givers of patients with co-morbid medical and psychological illnesses.

There were no overall increases in the size of professional networks for information sharing, referral or other forms of collaboration. Apparent decreases in the use of non-specified counsellors in favour of increased links with specific psychological services such as psychiatrists, psychologists and the Veterans and Veterans Families Counselling Service (VVCS) suggests a shift in practice towards the overt and deliberate prioritisation of mental health supports for this patient group, and a greater awareness of the role and utility of the VVCS, in keeping with the awareness-raising of this service incorporated into the program.

The workshops were ambitious in scope, and occasionally fell short in execution. We attended some workshops where almost all the attendees were doctors, with a few nurses, and others where psychologists were the largest single group. The content of the workshop sometimes struggled to be meaningful for specific health disciplines, rather than for a generic ‘health worker’ audience. Some psychologists expressed concern that the rapid overview of complex psychological interventions might engender in other participants over-confidence about their ability to deliver complex psychological interventions.

Our evaluation found an increase in the use of mindfulness and motivational interviewing, but not other strategies, among participants who were not mental health professionals. Neither of these strategies is new in the general practice therapeutic landscape. Motivational interviewing, in particular, forms the basis of standard general practice guidelines such as those for smoking cessation [30]. In the absence of a control group (unwieldy in the evaluation design for such a large, multi-centred program), it is possible that the increase in stated use may be due to relabelling. Some clinicians may have simply relabelled strategies they already used after recognising their own practices described at the workshop as a named psychological strategy. If this were the case, the pre-workshop low rates of usage may have been an underestimate.

An important limitation to this study is the low numbers at follow-up. Typically, longitudinal surveys used in health education and evaluation have significant attrition in response rates to repeated surveys [31, 32] and our evaluation is no different. The response rate at three months had decreased from 73 % (post-workshop survey) to 47 % of the relevant population. An additional methodological issue may have been respondent burden, which seemed to particularly impact on the social network survey, with 16 % of those who completed the pre-workshop survey and 15 % of those who completed the three month survey failing to complete the social network survey. Social network surveys are recognised as imposing significant respondent burden [33]. In our study the minimum number of items in the social network survey was 75 (15 names, 5 types of relationships), to a maximum of 90. We organised the survey by alters (asking all five questions about each alter, or health discipline) rather than by questions (asking one question at a time for all alters), due to the high number of alters we identified. For written surveys, response rates are higher if the surveys are organised by question rather than alter, probably because the appearance of respondent burden is reduced [34]. We also found that the unique identifier – a combination of middle name and year of birth was irregularly used, limiting our capacity to undertake paired analyses.

Although social network analysis is likely to be a valuable evaluation tool for interprofessional education, our study results suggest that the total number of names generated should be limited and should be arranged by question rather than alter. This holds true for surveys in both paper and web-based form. Additionally, resources should be specifically devoted to enhancing response – for example, through using computer assisted telephone calls, or by placing an incentive on return of survey (such as continuing education recognition).

The second reason for low response rates is the relatively lengthy roll-out of the program, as it was delivered through organisations across Australia. This meant that some of the participants received the training too late in the study period to participate in the formal three-month follow-up survey. We did not find any geographic bias in our study, and although the follow-up data overall includes a slight over representation of medical practitioners, the matched-pairs analysis enables this potential bias to be contained. Modelling for attrition bias in follow-up data suggest that attrition rates up to 60 % are acceptable if the missing cases are missing at random [30] Our attrition rate is around 69 % (74 % for social network data), and our survey is underpowered to detect subtle changes in social network characteristics. The responses from the three month survey may have been susceptible to respondent bias, in that those who felt most positively about the program may have been more likely to respond to repeated surveys. If this were the case, the level of improvement at three months in this study may be overstated.

Despite these limitations, our evaluation suggests that there were positive outcomes in relation to content knowledge, skills and collaborative networks through the use of inter-professional educational workshops addressing health care for primary care patients with co-morbid illnesses. The evidence for the effectiveness of interprofessional education on collaboration or teamwork has not to date been particularly strong for primary care settings [35]. It is worth noting therefore that particular structural drivers existed in the Australian primary care setting, which increased the likelihood of success of Mind the Gap. The changes that were sought, at least in general practice, were not complex, and could be readily incorporated into existing organisational and clinical work. The person-power to support enhanced case-finding now exists with the widespread uptake of practice nurses into general practice over the last decade [36]. Private health professionals other than GPs had structural incentives to collaborate with GPs, since GPs may refer patients with psychological and medical co-morbidities to private allied health professionals and psychologists for services fundable under the national insurer, Medicare. Mental health professionals in the public sector had an interest in reducing the size of their complex workload through better collaboration. Thus, there were few attendees at the workshops who could not identify immediate benefits to themselves arising from interprofessional collaboration for patients with psychological and medical co-morbidities.

## Conclusion

Simply placing health professionals in proximity to one another in an educational session is not sufficient to generate changes in attitude or collaborative practice. If run poorly, it may even cement prejudicial attitudes towards each other [37], Collaboration is a complex, polyfactorial organisational outcome. Education designers should be cognizant of local referral and collaborative networks, and structural drivers for collaboration, in order to identify and capitalise on effective drivers for change. Mind the Gap was “localised” – delivered by local facilitators, with local interprofessional participants, by the local support organisation - while also being framed by financial and organisational drivers to productive collaboration. A follow-up learning exercise that enabled the continuation of reflective engagement would have helped consolidate gains. Nevertheless, our evaluation suggests that there is likely to be value in educating groups of primary care professionals together on matters that are of direct clinical relevance to them, and for which there are existing structural drivers of collaboration.

## Additional files

Additional file 1: Figure S1. Pre-workshop Questionnaire, Mind the Gap Program Evaluation. Questionnaire delivered before the workshop. (PDF 79 kb) Additional file 2: Figure S2. Professional Network Questionnaire, Mind the Gap Program Evaluation. Questionnaire delivered before the workshop, and three months after the workshop (PDF 85 kb) Additional file 3: Table S1. Disciplines nominated as members of respondents’ professional networks. (DOCX 14 kb) Additional file 4: Figure S3. Post-workshop Questionnaire, Mind the Gap Program Evaluation. Questionnaire delivered at the conclusion of the Mind the Gap workshop. (PDF 81 kb) Additional file 5: Figure S4. Follow-up Questionnaire, Mind the Gap Program Evaluation. Questionnaire administered three months after completing Mind the Gap. (PDF 111 kb) Additional file 6: Table S2. Changes in size and characteristics of health providers’ networks (matched pairs analysis, *n* = 116). (DOCX 14 kb)
