# Supplementary material for: Impact of interprofessional education about psychological and medical comorbidities on practitioners’ knowledge and collaborative practice: mixed method evaluation of a national program
Source: BMC Health Serv Res. 2016 Sep 2;16(1):465. doi: 10.1186/s12913-016-1720-z (PMC5009489; doi:10.1186/s12913-016-1720-z)
Supplement: Additional file 1: Figure S1. — Pre-workshop Questionnaire, Mind the Gap Program Evaluation. Questionnaire delivered before the workshop. (PDF 79 kb) [file 12913_2016_1720_MOESM1_ESM.pdf]

Pre-Workshop Questionnaire
Mind the Gap Program Evaluation

THE AUSTRALIAN NATIONAL UNIVERSITY

Workshop location
Your discipline

Please write the first two letters of your first name followed by the first two letters of your surname

Questions about knowledge

How would you rate your knowledge, at this point in time, of:

1. The **aetiology, epidemiology, range and inter-relationships** of mental health issues and chronic health conditions as co-morbidities

1	2	3	4	5
very poor	*poor*	*fair*	*good*	*very good*

2. **Patient self-management approaches and strategies** in managing mental health issues and chronic health conditions as co-morbidities

1	2	3	4	5
very poor	*poor*	*fair*	*good*	*very good*

3. **Consumer and carer perspectives and experiences** of co-morbid psychological and physical health conditions

1	2	3	4	5
very poor	*poor*	*fair*	*good*	*very good*

4. **How to assess** co-morbid psychological and physical health conditions

1	2	3	4	5
very poor	*poor*	*fair*	*good*	*very good*

5. **Management planning** (including use of MBS item numbers and chronic disease management care plans) for co-morbid psychological and physical health conditions

1	2	3	4	5
very poor	*poor*	*fair*	*good*	*very good*

6. **Relapse prevention strategies planning** for co-morbid psychological and physical health conditions

1	2	3	4	5
very poor	*poor*	*fair*	*good*	*very good*

Questions about awareness

7. **How many patients** with significant co-morbid physical and mental health issues would you typically see in a week?

____

8. **How much of a problem** do you perceive co-morbid mental / physical health conditions are in your practice?

1	2	3	4	5
None at all	*insignificant*	*moderate*	*significant*	*extreme*

Questions about confidence

How confident do you feel about:

9. **Recognising patients** with co-morbid mental and physical health conditions?

1	2	3	4	5
Very unsure	*not confident*	*somewhat*	*confident*	*very confident*

10. **Meeting the needs of carers** of patients with co-morbid mental and physical health conditions?

1	2	3	4	5
Very unsure	*not confident*	*somewhat*	*confident*	*very confident*

11. Using **psycho-educational strategies**?

1	2	3	4	5
Very unsure	*not confident*	*somewhat*	*confident*	*very confident*

12. **Integrating** pharmacological and psychotherapeutic strategies?

1	2	3	4	5
Very unsure	*not confident*	*somewhat*	*confident*	*very confident*

Questions about use of skills & techniques

In the last month that you worked, how often did you use the following techniques with patients with mental and physical health co-morbidities:

13. **Motivational interviewing?**

1	2	3	4	5
never	*once or twice*	*3-5 times*	*6 -15 times*	*> 15 times*

14. **Behavioral activation (CBT)?**

1	2	3	4	5
never	*once or twice*	*3-5 times*	*6 -15 times*	*> 15 times*

15. **Solution-focused therapy?**

1	2	3	4	5
never	*once or twice*	*3-5 times*	*6 -15 times*	*> 15 times*

16. **Mindfulness based therapy?**

1	2	3	4	5
never	*once or twice*	*3-5 times*	*6 -15 times*	*> 15 times*

17. **Relaxation strategies?**

1	2	3	4	5
never	*once or twice*	*3-5 times*	*6 -15 times*	*> 15 times*

18. **Grief & loss counselling?**

1	2	3	4	5
never	*once or twice*	*3-5 times*	*6-15 times*	*> 15 times*

19. **Other** (please describe)____

1	2	3	4	5
never	*once or twice*	*3-5 times*	*6-15 times*	*> 15 times*

Questions about attitudes [1]

At this point in time, based on my participation in Mind the Gap, and thinking about the group of professionals I might collaborate with for patients with mental and physical health co-morbidities :

20. I feel comfortable initiating discussions about sharing responsibility for client care

1	2	3	4	5	6	7
Not at all						*to a very great extent*

21. I am comfortable engaging in shared decision making with clients

1	2	3	4	5	6	7
Not at all						*to a very great extent*

22. I feel comfortable clarifying misconceptions about the role of someone in my profession

1	2	3	4	5	6	7
Not at all						*to a very great extent*

23. I see myself as preferring to work on an inter-professional team

1	2	3	4	5	6	7
Not at all						*to a very great extent*

24. I am comfortable being the leader in a team situation

1	2	3	4	5	6	7
Not at all						*to a very great extent*

25. I feel confident taking on different roles in a team (ie., leader, participant)

1	2	3	4	5	6	7
Not at all						*to a very great extent*

26. I feel comfortable speaking out within the team when others are not keeping the client's best interests in mind

1	2	3	4	5	6	7
Not at all						*to a very great extent*

27. I believe that inter-professional practice is difficult to implement.

1	2	3	4	5	6	7
Not at all						*to a very great extent*

[1] *Adapted from the ISVS. King G, Shaw L, Orchard C, Miller S. (2010) The Interprofessional Socialization and Valuing Scale: A tool for evaluating the shift toward collaborative care approaches in health care settings. Work: 35 (2010) 77-85*
