# Supplementary material for: Impact of interprofessional education about psychological and medical comorbidities on practitioners’ knowledge and collaborative practice: mixed method evaluation of a national program
Source: BMC Health Serv Res. 2016 Sep 2;16(1):465. doi: 10.1186/s12913-016-1720-z (PMC5009489; doi:10.1186/s12913-016-1720-z)
Supplement: Additional file 2: Figure S2. — Professional Network Questionnaire, Mind the Gap Program Evaluation. Questionnaire delivered before the workshop, and three months after the workshop (PDF 85 kb) [file 12913_2016_1720_MOESM2_ESM.pdf]

Professional Network Questionnaire
Mind the Gap Program Evaluation

THE AUSTRALIAN NATIONAL UNIVERSITY

Workshop location	
Your discipline	

Please write the first two letters of your first name followed by the first two letters of your surname

All questions pertain to the last three months, related to dealing with patients or clients with co-morbid physical and mental health condition.

1. How many patients with co-morbid physical and mental health issues would you see in a typical week? ____

2. In relation to the following health professionals in your local area, please answer the following questions, using the table below:
   - Have you sought information from this type of professional about particular clients?
   - Have you provided information to this type of professional about particular clients?
   - Have you referred clients specifically to this type of professional?
   - Has this type of professional referred clients to you?
   - Have you worked together with this type of professional in other ways (eg., to solve problems for patients?)

**Professional**	***Sought information***	***Provided information***	***Referred to***	***Referral from***	***Worked together***
Practice Nurse	☐ never<br>☐ occasionally<br>☐ often	☐ never<br>☐ occasionally<br>☐ often	☐ never<br>☐ occasionally<br>☐ often	☐ never<br>☐ occasionally<br>☐ often	☐ never<br>☐ occasionally<br>☐ often
Community Health Nurse	☐ never<br>☐ occasionally<br>☐ often	☐ never<br>☐ occasionally<br>☐ often	☐ never<br>☐ occasionally<br>☐ often	☐ never<br>☐ occasionally<br>☐ often	☐ never<br>☐ occasionally<br>☐ often
Pharmacist	☐ never<br>☐ occasionally<br>☐ often	☐ never<br>☐ occasionally<br>☐ often	☐ never<br>☐ occasionally<br>☐ often	☐ never<br>☐ occasionally<br>☐ often	☐ never<br>☐ occasionally<br>☐ often
Physiotherapist	☐ never<br>☐ occasionally<br>☐ often	☐ never<br>☐ occasionally<br>☐ often	☐ never<br>☐ occasionally<br>☐ often	☐ never<br>☐ occasionally<br>☐ often	☐ never<br>☐ occasionally<br>☐ often
Occupational therapist	☐ never<br>☐ occasionally<br>☐ often	☐ never<br>☐ occasionally<br>☐ often	☐ never<br>☐ occasionally<br>☐ often	☐ never<br>☐ occasionally<br>☐ often	☐ never<br>☐ occasionally<br>☐ often
Exercise physiologist	☐ never<br>☐ occasionally<br>☐ often	☐ never<br>☐ occasionally<br>☐ often	☐ never<br>☐ occasionally<br>☐ often	☐ never<br>☐ occasionally<br>☐ often	☐ never<br>☐ occasionally<br>☐ often

**Professional**	***Sought information***	***Provided information***	***Referred to***	***Referral from***	***Worked together***
Psychologist	☐ never<br>☐ occasionally<br>☐ often	☐ never<br>☐ occasionally<br>☐ often	☐ never<br>☐ occasionally<br>☐ often	☐ never<br>☐ occasionally<br>☐ often	☐ never<br>☐ occasionally<br>☐ often
VVCS (Veterans & Veterans Families Counseling Service)	☐ never<br>☐ occasionally<br>☐ often	☐ never<br>☐ occasionally<br>☐ often	☐ never<br>☐ occasionally<br>☐ often	☐ never<br>☐ occasionally<br>☐ often	☐ never<br>☐ occasionally<br>☐ often
Other counsellor	☐ never<br>☐ occasionally<br>☐ often	☐ never<br>☐ occasionally<br>☐ often	☐ never<br>☐ occasionally<br>☐ often	☐ never<br>☐ occasionally<br>☐ often	☐ never<br>☐ occasionally<br>☐ often
Psychiatrist	☐ never<br>☐ occasionally<br>☐ often	☐ never<br>☐ occasionally<br>☐ often	☐ never<br>☐ occasionally<br>☐ often	☐ never<br>☐ occasionally<br>☐ often	☐ never<br>☐ occasionally<br>☐ often
Cardiologist	☐ never<br>☐ occasionally<br>☐ often	☐ never<br>☐ occasionally<br>☐ often	☐ never<br>☐ occasionally<br>☐ often	☐ never<br>☐ occasionally<br>☐ often	☐ never<br>☐ occasionally<br>☐ often
Endocrinologist	☐ never<br>☐ occasionally<br>☐ often	☐ never<br>☐ occasionally<br>☐ often	☐ never<br>☐ occasionally<br>☐ often	☐ never<br>☐ occasionally<br>☐ often	☐ never<br>☐ occasionally<br>☐ often
Respiratory physician	☐ never<br>☐ occasionally<br>☐ often	☐ never<br>☐ occasionally<br>☐ often	☐ never<br>☐ occasionally<br>☐ often	☐ never<br>☐ occasionally<br>☐ often	☐ never<br>☐ occasionally<br>☐ often
Health educator	☐ never<br>☐ occasionally<br>☐ often	☐ never<br>☐ occasionally<br>☐ often	☐ never<br>☐ occasionally<br>☐ often	☐ never<br>☐ occasionally<br>☐ often	☐ never<br>☐ occasionally<br>☐ often
Alternative therapist	☐ never<br>☐ occasionally<br>☐ often	☐ never<br>☐ occasionally<br>☐ often	☐ never<br>☐ occasionally<br>☐ often	☐ never<br>☐ occasionally<br>☐ often	☐ never<br>☐ occasionally<br>☐ often
Other (please specify)<br>________________	☐ never<br>☐ occasionally<br>☐ often	☐ never<br>☐ occasionally<br>☐ often	☐ never<br>☐ occasionally<br>☐ often	☐ never<br>☐ occasionally<br>☐ often	☐ never<br>☐ occasionally<br>☐ often
Other (please specify)<br>________________	☐ never<br>☐ occasionally<br>☐ often	☐ never<br>☐ occasionally<br>☐ often	☐ never<br>☐ occasionally<br>☐ often	☐ never<br>☐ occasionally<br>☐ often	☐ never<br>☐ occasionally<br>☐ often
Other (please specify)<br>________________	☐ never<br>☐ occasionally<br>☐ often	☐ never<br>☐ occasionally<br>☐ often	☐ never<br>☐ occasionally<br>☐ often	☐ never<br>☐ occasionally<br>☐ often	☐ never<br>☐ occasionally<br>☐ often

Thank you for your time
