# Supplementary material for: Impact of interprofessional education about psychological and medical comorbidities on practitioners’ knowledge and collaborative practice: mixed method evaluation of a national program
Source: BMC Health Serv Res. 2016 Sep 2;16(1):465. doi: 10.1186/s12913-016-1720-z (PMC5009489; doi:10.1186/s12913-016-1720-z)
Supplement: Additional file 3: Table S1. — Disciplines nominated as members of respondents’ professional networks. (DOCX 14 kb) [file 12913_2016_1720_MOESM3_ESM.docx]

**TABLE 1: Disciplines nominated as members of respondents’ professional networks**

| **Allied health** | Pharmacists*  Exercise physiologists*  Dieticians  Podiatrists  Social workers  Optometrists  Audiologists  Occupational therapists*  Physiotherapists*  Health educators (generic)*  Diabetes educators |
| --- | --- |
| **Community services** | Community nursing*  Aboriginal health workers  Drug & Alcohol services  Sexual Assault services  Domestic Violence services  Probation & Parole  Student & course-related counselling services  Community / welfare workers  Palliative care  Youth workers  Infant welfare nurse  Nurse practitioners |
| **Complementary and alternative therapists** | Alternative therapists (generic)*  Osteopaths  Chiropractors  Yoga teachers  Acupuncturists |
| **General practice** | General practitioners  Practice nurses*  Reception staff |
| **Medical specialists** | Cardiologists*  Endocrinologists*  Respiratory Physicians*  Other medical specialists |
| **Mental health** | Psychologists*  Veterans and Veterans’ Families Counselling Service*  Psychiatrists*  Mental health nursing  Other counsellors*  Mental Health crisis team  Mental Health Social Worker |
| **Specialised services** | Clergy/Pastoral counsellors  Day activities centre  Interpreters  Wellness centre or coaches  Bereavement counsellors  Centrelink workers  Work-related services  Family members/ carers |

**Included as one of the 15 set options in the social network survey; remainder nominated as one of the 6 free options in the survey*
