# Supplementary material for: Impact of interprofessional education about psychological and medical comorbidities on practitioners’ knowledge and collaborative practice: mixed method evaluation of a national program
Source: BMC Health Serv Res. 2016 Sep 2;16(1):465. doi: 10.1186/s12913-016-1720-z (PMC5009489; doi:10.1186/s12913-016-1720-z)
Supplement: Additional file 4: Figure S3. — Post-workshop Questionnaire, Mind the Gap Program Evaluation. Questionnaire delivered at the conclusion of the Mind the Gap workshop. (PDF 81 kb) [file 12913_2016_1720_MOESM4_ESM.pdf]

# Post Workshop Questionnaire
## Mind the Gap Program Evaluation

THE AUSTRALIAN NATIONAL UNIVERSITY

| | |
|---|---|
| *Workshop location* | |
| *Your discipline* | |

*Please write the first two letters of your first name followed by the first two letters of your surname*

| | | | |
|---|---|---|---|
| | | | |

### Questions about knowledge

*How would you rate your knowledge, at this point in time, of:*

1. The **aetiology, epidemiology, range and inter-relationships** of mental health issues and chronic health conditions as co-morbidities

| 1 | 2 | 3 | 4 | 5 |
|---|---|---|---|---|
| *very poor* | *poor* | *fair* | *good* | *very good* |

2. **Patient self-management approaches and strategies** in managing mental health issues and chronic health conditions as co-morbidities

| 1 | 2 | 3 | 4 | 5 |
|---|---|---|---|---|
| *very poor* | *poor* | *fair* | *good* | *very good* |

3. **Consumer and carer perspectives and experiences** of co-morbid psychological and physical health conditions

| 1 | 2 | 3 | 4 | 5 |
|---|---|---|---|---|
| *very poor* | *poor* | *fair* | *good* | *very good* |

4. **How to assess** co-morbid psychological and physical health conditions

| 1 | 2 | 3 | 4 | 5 |
|---|---|---|---|---|
| *very poor* | *poor* | *fair* | *good* | *very good* |

5. **Management planning** (including use of MBS item numbers and chronic disease management care plans) for co-morbid psychological and physical health conditions

| 1 | 2 | 3 | 4 | 5 |
|---|---|---|---|---|
| *very poor* | *poor* | *fair* | *good* | *very good* |

6. **Relapse prevention strategies planning** for co-morbid psychological and physical health conditions

| 1 | 2 | 3 | 4 | 5 |
|---|---|---|---|---|
| *very poor* | *poor* | *fair* | *good* | *very good* |

## Questions about confidence

*How confident do you feel, at this point in time, in:*

7. **Recognising patients** with co-morbid psychological and physical health conditions?

| 1 | 2 | 3 | 4 | 5 |
|---|---|---|---|---|
| *Very unsure* | *not confident* | *somewhat* | *confident* | *very confident* |

8. **Meeting the needs of carers** of patients with co-morbid psychological and physical health conditions?

| 1 | 2 | 3 | 4 | 5 |
|---|---|---|---|---|
| *Very unsure* | *not confident* | *somewhat* | *confident* | *very confident* |

9. Using **psycho-educational** strategies?

| 1 | 2 | 3 | 4 | 5 |
|---|---|---|---|---|
| *Very unsure* | *not confident* | *somewhat* | *confident* | *very confident* |

10. **Integrating pharmacological** and psychotherapeutic strategies?

| 1 | 2 | 3 | 4 | 5 |
|---|---|---|---|---|
| *Very unsure* | *not confident* | *somewhat* | *confident* | *very confident* |

## Questions about plans for change

*Please list three (3) specific changes you intend to make in your practice relating to clients with co-morbid psychological and mental health conditions:*

1. ______________________________

How committed are you to this change? ☐ *Highly* ☐ *Moderately* ☐ *Slightly*

2. ______________________________

How committed are you to this change? ☐ *Highly* ☐ *Moderately* ☐ *Slightly*

3. ______________________________

How committed are you to this change? ☐ *Highly* ☐ *Moderately* ☐ *Slightly*
