# Supplementary material for: Impact of interprofessional education about psychological and medical comorbidities on practitioners’ knowledge and collaborative practice: mixed method evaluation of a national program
Source: BMC Health Serv Res. 2016 Sep 2;16(1):465. doi: 10.1186/s12913-016-1720-z (PMC5009489; doi:10.1186/s12913-016-1720-z)
Supplement: Additional file 5: Figure S4. — Follow-up Questionnaire, Mind the Gap Program Evaluation. Questionnaire administered three months after completing Mind the Gap. (PDF 111 kb) [file 12913_2016_1720_MOESM5_ESM.pdf]

Follow Up Questionnaire
Mind the Gap Program Evaluation

THE AUSTRALIAN NATIONAL UNIVERSITY

Workshop location
Your discipline

Please write the first two letters of your first name followed by the first two letters of your surname

Questions about knowledge

How would you rate your knowledge, at this point in time, of:

1. The **aetiology, epidemiology, range and inter-relationships** of mental health issues and chronic health conditions as co-morbidities

1	2	3	4	5
very poor	*poor*	*fair*	*good*	*very good*

2. **Patient self-management approaches and strategies** in managing mental health issues and chronic health conditions as co-morbidities

1	2	3	4	5
very poor	*poor*	*fair*	*good*	*very good*

3. **Consumer and carer perspectives and experiences** of co-morbid psychological and physical health conditions

1	2	3	4	5
very poor	*poor*	*fair*	*good*	*very good*

4. **How to assess** co-morbid psychological and physical health conditions

1	2	3	4	5
very poor	*poor*	*fair*	*good*	*very good*

5. **Management planning** (including use of MBS item numbers and chronic disease management care plans) for co-morbid psychological and physical health conditions

1	2	3	4	5
very poor	*poor*	*fair*	*good*	*very good*

6. **Relapse prevention strategies planning** for co-morbid psychological and physical health conditions

1	2	3	4	5
very poor	*poor*	*fair*	*good*	*very good*

Questions about awareness

7. **How many patients** with significant co-morbid physical and mental health issues would you typically see in a week?

____

8. **How much of a problem** are co-morbid mental / physical health conditions in your practice?

1	2	3	4	5
Not at all	*insignificant*	*moderate*	*significant*	*extreme*

Questions about confidence

How confident do you feel about:

9. **Recognising patients** with co-morbid mental and physical health conditions?

1	2	3	4	5
Very unsure	*not confident*	*somewhat*	*confident*	*very confident*

10. **Meeting the needs of carers** of patients with co-morbid mental and physical health conditions?

1	2	3	4	5
Very unsure	*not confident*	*somewhat*	*confident*	*very confident*

11. Using **psycho-educational strategies**?

1	2	3	4	5
Very unsure	*not confident*	*somewhat*	*confident*	*very confident*

12. **Integrating** pharmacological and psychotherapeutic strategies?

1	2	3	4	5
Very unsure	*not confident*	*somewhat*	*confident*	*very confident*

Questions about use of skills & techniques

In the last month that you worked, how often did you use the following techniques with patients with mental and physical health co-morbidities:

13. **Motivational interviewing?**

1	2	3	4	5
never	*once or twice*	*3-5 times*	*6 -15 times*	*> 15 times*

14. **Behavioral activation (CBT)?**

1	2	3	4	5
never	*once or twice*	*3-5 times*	*6 -15 times*	*> 15 times*

15. **Solution-focused therapy?**

1	2	3	4	5
never	*once or twice*	*3-5 times*	*6 -15 times*	*> 15 times*

16. **Mindfulness based therapy?**

1	2	3	4	5
never	*once or twice*	*3-5 times*	*6 -15 times*	*> 15 times*

17. **Relaxation strategies?**

1	2	3	4	5
never	*once or twice*	*3-5 times*	*6 -15 times*	*> 15 times*

18. **Grief & loss counselling?**

1	2	3	4	5
never	*once or twice*	*3-5 times*	*6-15 times*	*> 15 times*

19. **Other** (please describe)____

1	2	3	4	5
never	*once or twice*	*3-5 times*	*6-15 times*	*> 15 times*

Questions about attitudes [1]

At this point in time, based on my participation in Mind the Gap, and thinking about the group of professionals I might collaborate with for patients with mental and physical health co-morbidities :

20. I feel comfortable initiating discussions about sharing responsibility for client care

1	2	3	4	5	6	7
Not at all						*to a very great extent*

21. I am comfortable engaging in shared decision making with clients

1	2	3	4	5	6	7
Not at all						*to a very great extent*

22. I feel comfortable clarifying misconceptions about the role of someone in my profession

1	2	3	4	5	6	7
Not at all						*to a very great extent*

23. I see myself as preferring to work on an inter-professional team

1	2	3	4	5	6	7
Not at all						*to a very great extent*

24. I am comfortable being the leader in a team situation

1	2	3	4	5	6	7
Not at all						*to a very great extent*

25. I feel confident taking on different roles in a team (ie., leader, participant)

1	2	3	4	5	6	7
Not at all						*to a very great extent*

26. I feel comfortable speaking out within the team when others are not keeping the client's best interests in mind

1	2	3	4	5	6	7
Not at all						*to a very great extent*

27. I believe that inter-professional practice is difficult to implement.

1	2	3	4	5	6	7
Not at all						*to a very great extent*

[1] *Adapted from the ISVS. King G, Shaw L, Orchard C, Miller S. (2010) The Interprofessional Socialization and Valuing Scale: A tool for evaluating the shift toward collaborative care approaches in health care settings. Work: 35 (2010) 77-85*

Questions about changes to your practice

	Change 1	Change 2	Change 3
Please list three (3) specific changes you have made in your practice relating to clients with co-morbid physical and psychological health conditions in the last 3 months:	____________________<br>____________________<br>____________________<br>____________________	____________________<br>____________________<br>____________________<br>____________________	____________________<br>____________________<br>____________________<br>____________________
Is this the same change you intended to make at the end of the Mind the Gap workshop?	□ Yes<br>□ No<br>□ Not sure	□ Yes<br>□ No<br>□ Not sure	□ Yes<br>□ No<br>□ Not sure
If not, what is different & why?	____________________<br>____________________<br>____________________<br>____________________	____________________<br>____________________<br>____________________<br>____________________	____________________<br>____________________<br>____________________<br>____________________
How difficult was it to make this change?	1 Very difficult<br>2 Difficult<br>3 Neutral<br>4 Easy<br>5 Very easy	1 Very difficult<br>2 Difficult<br>3 Neutral<br>4 Easy<br>5 Very easy	1 Very difficult<br>2 Difficult<br>3 Neutral<br>4 Easy<br>5 Very easy
Do you intend to continue with this change?	□ Yes<br>□ No<br>□ Not sure	□ Yes<br>□ No<br>□ Not sure	□ Yes<br>□ No<br>□ Not sure
