# Supplementary material for: Impact of interprofessional education about psychological and medical comorbidities on practitioners’ knowledge and collaborative practice: mixed method evaluation of a national program
Source: BMC Health Serv Res. 2016 Sep 2;16(1):465. doi: 10.1186/s12913-016-1720-z (PMC5009489; doi:10.1186/s12913-016-1720-z)
Supplement: Additional file 6: Table S2. — Changes in size and characteristics of health providers’ networks (matched pairs analysis, n = 116). (DOCX 14 kb) [file 12913_2016_1720_MOESM6_ESM.docx]

**TABLE 2. Changes in size and characteristics of health providers’ networks (matched pairs analysis, n=116)**

| ***Health professional network in preceding three months*** | ***Mean at baseline*** | ***Mean at three months*** |
| --- | --- | --- |
| Number of health disciplines that practitioner has refereed to | 8.10 | 8.72 |
| Number of health disciplines that have referred to practitioner | 6.07 | 6.12 |
| Frequency of referral to different health disciplines | 11.23 | 12.17^[[1]](#footnote-1)^ |
| Frequency of referral from different health disciplines | 8.10 | 8.13 |
| Number of health disciplines that practitioner gave information to | 7.98 | 8.751 |
| Number of health disciplines practitioners asked for information | 7.97 | 8.47 |
| Frequency of sharing information with different health disciplines | 10.85 | 11.06 |
| Frequency of other health disciplines sharing information with practitioner | 10.91 | 11.39 |
| Mean size of information exchange network | 9.92 | 9.78 |
| Mean size of referral network | 9.76 | 9.62 |
| Mean size of collaboration network | 8.03 | 7.79 |

1 p<0.05

1. [↑](#footnote-ref-1)
